# Supplementary material for: AlphaFind v2: similarity search in AlphaFold DB and TED domains across structural contexts
Source: Nucleic Acids Res. 2026 Apr 24;54(W1):W206–10. doi: 10.1093/nar/gkag372 (PMC13355127; doi:10.1093/nar/gkag372)
Supplement: gkag372_Supplemental_File [file gkag372_supplemental_file.pdf]

Supplementary Materials: AlphaFind v2: Similarity Search in AlphaFold DB and TED Domains across Structural Contexts

Supplementary Text S1: Workflow - TED domains

In the TED domains search mode, the query and the candidates are protein domains. In the TED Multidomain mode, the results of search for all the query domains are merged. For each target, a combined, altered TM-score is computed:

$$TM_{\text{target}} = \frac{1}{N_{\text{target}}} \sum_{i=1}^{N_{\text{common}}} \frac{1}{1 + d_i^2} \tag{1}$$

where N_{target} is the number of domains in the target protein (TM_{query} can be defined symmetrically), N_{common} is the number of domains which have their matching counterparts in both query and target, and d_i is RMSD between the i-th pair of matching query-target domains after their independent alignment. This TM-score behaves as one would expect – it grows higher with better match of individual domains as well as with more domains considered.

Supplementary Text S2: Performance Evaluation

This supplementary material extends the *Performance* Section of the main paper text. The input data, scripts to reproduce the experiments as well as the raw and the aggregated results are available and further documented at `https://doi.org/10.6084/m9.figshare.31802743`.

Dataset

The dataset used for performance evaluation is taken from [2] (referred to as *AF23M*), also available available for download at `https://doi.org/10.6084/m9.figshare.30546650.v1`.

The dataset contains a listing of protein chain identifiers, which contain two or more high-confidence domains. In total, the dataset contains 2050 protein chains and 4420 domains from 1038 unique CATH families. Most of the proteins chains contain two domains (82.9%), followed by chains with three domains (14.8%) and the rest (2.3%) containing four or five domains. Figure S1 supplements these statistics with histograms of the number of atoms and residues in the chains and domains.

Evaluation pipeline

The evaluation design focused on the performance of AlphaFind v2 compared with other structural similarity search services with publicly available API endpoints. The evaluation considers two metrics – the *time* it takes to get the search results and structural similarity to the input chain or domain measured by *TM-Score* [3].

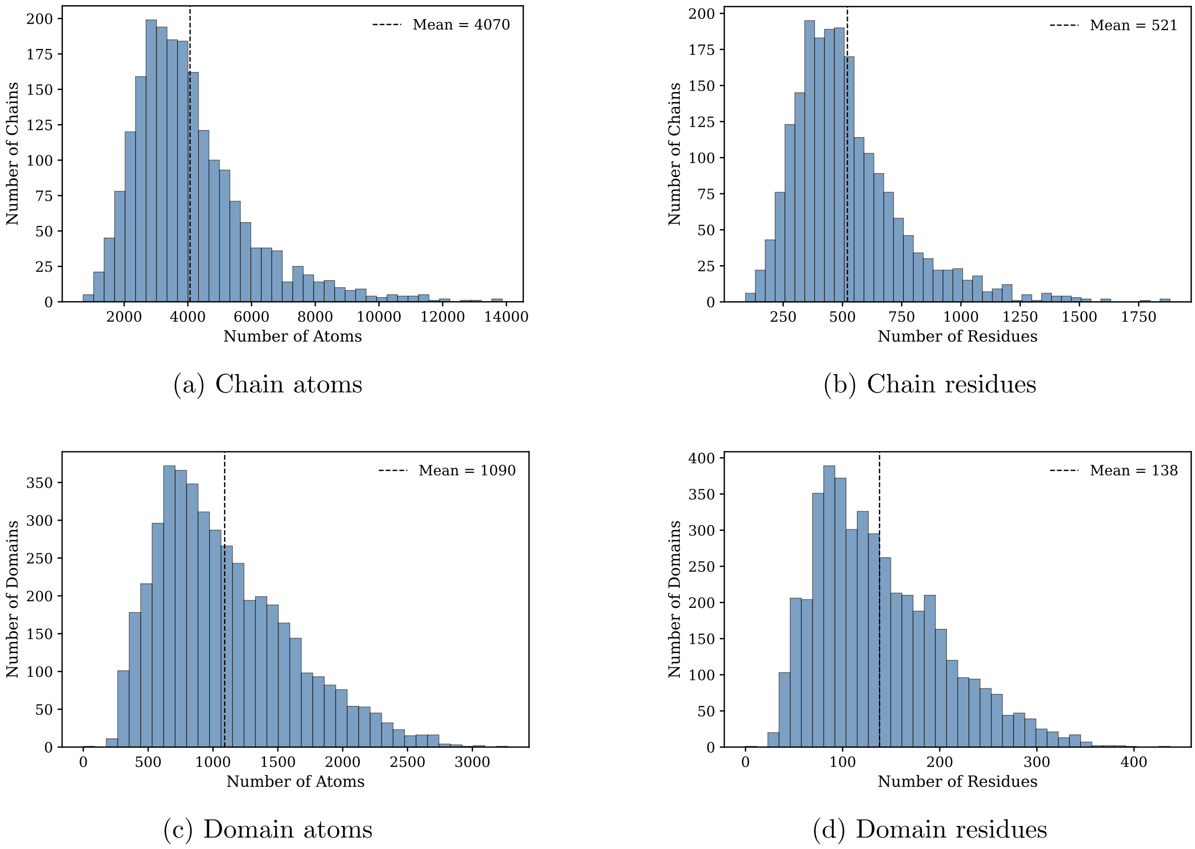

(a) Chain atoms (b) Chain residues

(c) Domain atoms (d) Domain residues

Figure S1: Histogram of the number of atoms and residues of the used dataset.

Evaluation of protein chains

The evaluation pipeline begins by downloading the needed data: a protein chain list from the figshare repository of [2], followed by downloading the corresponding raw PDB structures from the AlphaFold DB (`https://alphafold.ebi.ac.uk/`) API. Each structure is subsequently submitted to the FoldSeek Server API for search against the *'afbd-50'* database. Because FoldSeek does not provide TM-Scores, these are computed post hoc using USalign (`https://github.com/pylelab/USalign`). This computation is excluded from the reported search time. As FoldSeek returns a variable number of hits per query the result count for each query is recorded and used to parameterize subsequent searches, ensuring consistent comparison. Consequently, FoldSeek must be executed first in the pipeline.

Search performance is then evaluated for AlphaFind v1. Each query includes a UniProt identifier and a result limit equal to the corresponding FoldSeek output size. The script polls the API until results are available and records total latency from submission to completion. TM-scores are returned directly by the API and require no additional processing.

For AlphaFind v2, the API provides two result stages: approximate results based on embedding similarity and final results with computed TM-scores. Queries are submitted with a parameter matching the FoldSeek-derived result count. Two timing metrics are captured: (i) approximate time, defined as the interval from submission to retrieval of all kNN-based scores, and (ii) TM-score time, defined as the interval until all exact TM-scores are available.

All outputs are stored as per-query JSON files containing metadata, ranked results, TM-scores, and timing data. FoldSeek outputs are additionally exported as CSV files following TM-score computation. A final aggregation step processes all results, computing mean TM-scores across queries at multiple top-N thresholds and producing a summary CSV with per-method statistics, including average result counts, TM-score metrics, and timing measurements.

Protein domains

In protein domains, the evaluation flow follows a similar pattern to chains. The download script again uses the listing from [2] to fetch the domain structures PDB files from The Encyclopedia of Domains (`https://ted.cathdb.info/`) API.

Search performance evaluation starts with Merizo search, using the Psipred search API (`https://bioinf.cs.ucl.ac.uk/psipred/api`). The service returns the results with TM-Scores and other metadata (CATH annotation, taxonomy, etc.). Similar to FoldSeek, Merizo returns a variable number of results, therefore it needs to be executed first.

For AlphaFind v2 domains, the process mirrors the chains workflow described earlier: queries specify the domain identifier, the same k parameter matching mechanism is used to match Merizo's result counts, and the same two timing metrics are recorded – approximate time for initial results and TM-Score time for exact computation.

The raw results follow the same storage format as chains (JSON for AlphaFind v2, TSV for Merizo). The aggregation script handles both result types separately from chains, computing domain-specific mean TM-Scores and timing statistics before combining them into a domains summary CSV alongside the chains summary.

Results

As reported in Table 1 of the main text, AlphaFind v2 shows the best overall performance in the mean TM-Scores of its results. Statistical testing confirms that these differences are significant. Paired tests on protein chains show that AlphaFind v2 outperforms AlphaFind v1 ($p < 10^{-94}$) and FoldSeek Server ($p \approx 0$). Figure S2 shows the TM-Score distribution of the evaluated methods. Note, that it is important to interpret the TM-Score comparison of AlphaFind v2 and FoldSeek in the context of the caveats described in the following Section.

In terms of speed, AlphaFind v2 provides the fastest approximate results (*"a"*) (see Table 1 in the main text). As shown in Figure S3, its total runtime (*"tm"*) is comparable to FoldSeek Server and substantially lower than AlphaFind v1, demonstrating improved efficiency without loss of accuracy relative to its predecessor.

In the domain-level task, AlphaFind v2 again achieves the higher accuracy (0.947 / 0.894) and also reduces runtime by approximately four- to fivefold. Independent tests on domain-level data further demonstrate a significant improvement over Merizo-search ($p < 10^{-130}$).

Overall, AlphaFind v2 offers a consistent improvement in alignment quality while maintaining or reducing computational cost relative to baseline methods.

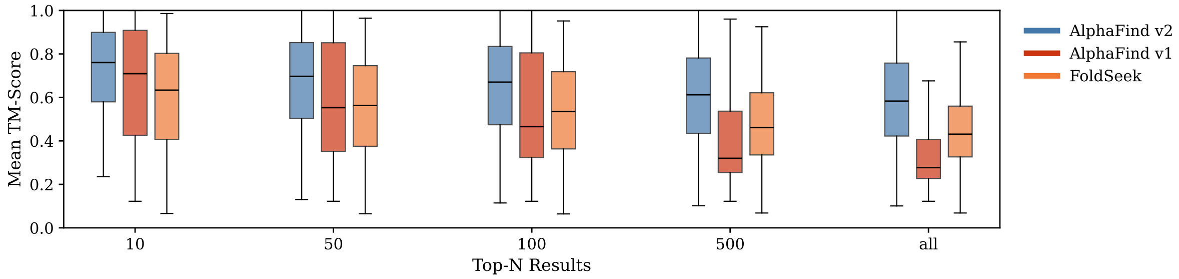

(a) Chains

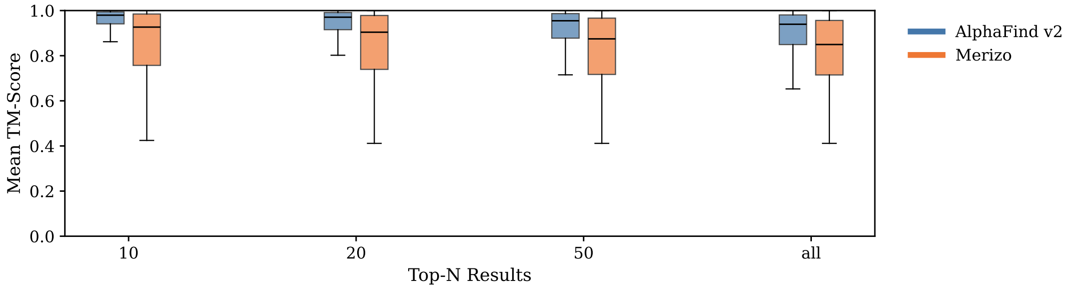

(b) Domains

Figure S2: TM-Score ranges of all methods in both search setups.

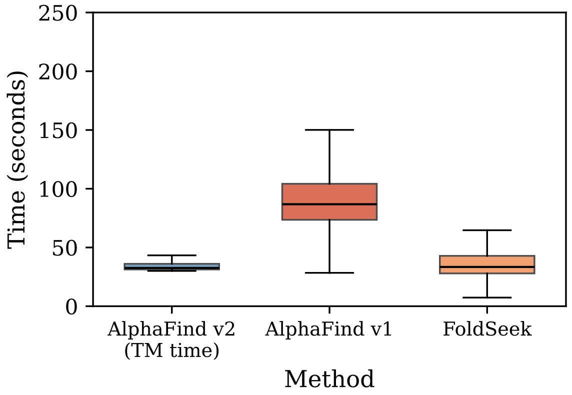

(a) Chains

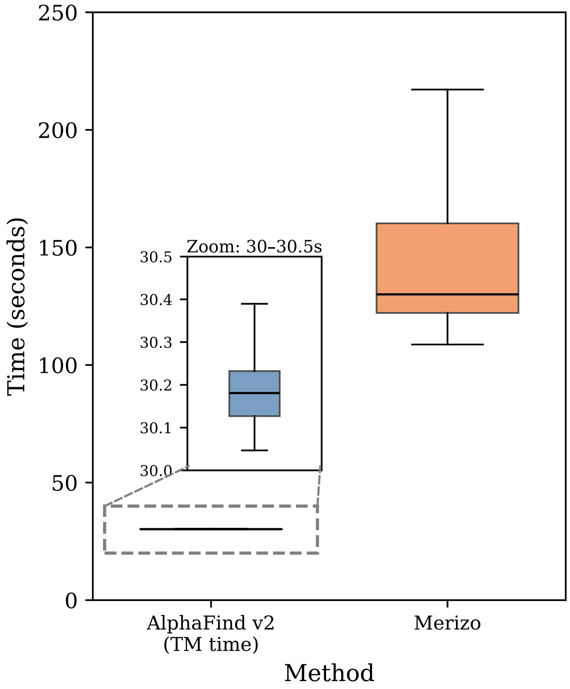

(b) Domains

Figure S3: Runtime ranges of all methods in both search setups. For AlphaFold v2, only the *"tm"* time is shown.

Limitations

The comparison between FoldSeek and AlphaFind v2 is inherently constrained by differences in the underlying target databases, which vary both in version and in preprocessing.

For predicted structures, FoldSeek Server uses the *afdb-50* database derived from AlphaFold DB (AFDB) v6, whereas AlphaFind v2 uses AFDB v4. Although these releases share substantial overlap, AFDB v6 is an expanded set of v4 data, incorporating 65.7 million additions, 39.3 million deletions, and 276.5 thousand modifications [1].

FoldSeek also operates on a version of AFDB clustered at 50% sequence identity, reducing redundancy but increasing structural diversity within the retained representatives. As such, the target database is approximately 52 million structures in size [1] [2], while AlphaFind v2 operates with the full AFDB v4 data (214 million structures). **We expect that this difference biases structural similarity metrics negatively for FoldSeek as near-identical chains are not included in the FoldSeek result set.**

We acknowledge that these discrepancies limit strict comparability; however, the analysis focuses solely on the proportion of the returned structural hits, rather than on direct, structure-level correspondence between returned results.

AlphaFind v1 uses AFDB v3 data. The transition from AFDB v3 to v4 introduced comparatively modest changes, primarily consisting of incremental additions of new proteomes and limited updates to existing predictions. As such, the comparison should not be limited by this. Both services use the 214-million set.

References

[1] Inigo Barrio-Hernandez et al. Clustering predicted structures at the scale of the known protein universe. *Nature*, 622:637–645, 2023. [PubMed:37704730][PubMed Central:PMC10584675][doi:10.1038/s41586-023-06510-w].

[2] Joan Segura, Ruben Sanchez-Garcia, Sebastian Bittrich, Yana Rose, Stephen K Burley, and Jose M Duarte. Multi-scale structural similarity embedding search across entire proteomes. *Bioinformatics*, 2026. [doi:10.1093/bioinformatics/btag058].

[3] Yang Zhang and Jeffrey Skolnick. Scoring function for automated assessment of protein structure template quality. *Proteins*, 57(4):702–710, 2004. [PubMed:15476259][doi:10.1002/prot.20264].

[1] Based on the reports from `https://ftp.ebi.ac.uk/pub/databases/alphafold/README.txt`

[2] We were unable to verify the exact number for AFDB v6 data, this is an estimate from the clustering FoldSeek previously reported for the AFDB v4 data.
